# Is Left Ventricular Global Longitudinal Strain by Two-Dimensional Speckle Tracking Echocardiography in Sepsis Cardiomyopathy Ready for Prime Time Use in the ICU?

**DOI:** 10.3390/healthcare7010005

**Published:** 2019-01-03

**Authors:** Venu Madhav Velagapudi, Rahul Pidikiti, Dennis A. Tighe

**Affiliations:** 1 Division of Pulmonary and Critical Care Medicine, Yale University School of Medicine, New Haven, CT 06510, USA; 2 Division of Cardiovascular Medicine, University of Massachusetts Medical School, Worcester, MA 01655, USA; rahulpidikiti@gmail.com (R.P.); dennis.tighe@umassmemorial.org (D.A.T.)

**Keywords:** sepsis cardiomyopathy, left ventricular function, global longitudinal strain

## Abstract

Myocardial deformation imaging (strain imaging) is a technique to directly quantify the extent of myocardial contractility and overcomes several of the limitations of ejection fraction. The application of the most commonly used strain imaging method; speckle-tracking echocardiography to patients with sepsis cardiomyopathy heralds an exciting development to the field. However; the body of evidence and knowledge on the utility, feasibility and prognostic value of left ventricular global longitudinal strain in sepsis cardiomyopathy is still evolving. We conducted a review of literature on utility of left ventricular global longitudinal strain in sepsis cardiomyopathy. We discuss the role of left ventricular global longitudinal strain in mortality prediction, utility and limitations of the technique in the context of sepsis cardiomyopathy.

## 1. Introduction

Left ventricular (LV) function is a powerful predictor of prognosis in a number of conditions and has been shown specifically to be predictive of outcomes in sepsis [1]. Sepsis cardiomyopathy, the reversible myocardial depression that occurs early in severe sepsis and septic shock was first described in 1970s [2]. Utilizing radionuclide angiography, Parker et al. [2] reported that 50% of patients with septic shock had severely reduced baseline LV ejection fraction which was paradoxically lower in survivors. An accepted definition of sepsis cardiomyopathy is based on an LV ejection fraction of less than 45% to 50% in the absence of previously diagnosed cardiac disease that demonstrates reversibility upon remission in patients without prior cardiomyopathy [3]. This definition was evolved prior to the availability of echocardiographic techniques such as speckle tracking echocardiography [4].

The traditional method used to assess LV function (in the ICU) has been determination of LV ejection fraction, usually based on visual analysis of two-dimensional (2D) images or Simpson biplane method [5]. This long relied-upon parameter to describe LV systolic function is relatively easy to acquire and is a concept familiar to most clinicians. However, significant limitations of using LV ejection fraction to characterize systolic function are recognized. The use of 2D echocardiography to describe cardiac function is influenced by geometric assumptions, and technical issues, such as apical foreshortening and difficulties in proper delineation of the endocardial borders, limit its accuracy. As a parameter to assess LV function, ejection fraction is highly dependent on loading conditions and as such does not directly reflect the underlying lying state of LV myocardial contractility. In addition, the reproducibility of this method is quite high with significant inter-observer variability reported [6,7,8].

Given these limitations, a method that more directly assesses intrinsic myocardial contractility would be desired for clinical use. Myocardial deformation imaging (also known as strain imaging) provides a means to directly quantify the extent of myocardial contractility and overcome several of the limitations of using ejection fraction for this purpose. Strain, a unit-less parameter, is defined as the percentage change in the length (deformation) of a myocardial segment over a given period of time compared to the resting state. The most widely used method to perform strain imaging is speckle-tracking echocardiography, a technique which makes use of the presence of unique acoustic markers (“speckles”) within the myocardium to track their position throughout the cardiac cycle. This method offers distinct advantages in comparison to earlier (and now rarely-used) Doppler-based techniques [9] and is now available on most current generation echocardiography platforms. Strain can be assessed in 3 principle directions (longitudinal, circumferential, and radial), however longitudinal strain is the most reproducible. Furthermore, as global strain has much better reproducibility than segmental strains, it is currently recommended that global longitudinal strain (GLS) be the parameter used to describe LV systolic function [5]. In an effort to provide some guidance, the most recent recommendation from the American Society of Echocardiography (ASE) and the European Association of Cardiovascular Imaging (EACVI) states that a peak GLS in the range −20% can be expected in a healthy person.

Strain-imaging by speckle-tracking echocardiography has been shown to have clinical utility in a variety of settings [9] and to offer superior prognostic value to ejection fraction for predicting major adverse cardiac events [10]. Advantages of using GLS to assess LV systolic function compared to ejection fraction include better reproducibility, ability to identify sub-clinical LV dysfunction, non-reliance on geometric assumptions, and lack of influence by tethering effects.

As the utility of GLS measurement by speckle tracing echocardiography has shown accuracy in predicting outcomes in several pathological conditions, it is logical to examine the role of GLS by speckle tracking 2D echocardiography in ICU patients with sepsis and sepsis cardiomyopathy.

Since GLS is most reproducible and commonly used strain parameter, we sought to review the current literature on role of GLS in sepsis cardiomyopathy with a focus on current limitations, pitfalls of strain acquisition, standardization and clinical relevance towards mortality prediction.

## 2. Materials and Methods

We conducted a review of current literature on the utility and prognostic value of left ventricular global longitudinal strain in patients with sepsis cardiomyopathy.

We have conducted a systematic search of PubMed data search for (((((sepsis OR septic)) AND (cardiac output OR echo OR TTE)) AND (heart diseases/etiology OR heart assist devices OR heart failure OR dysfunction OR ejection factor)) AND (strain) OR (speckle)) from January 1976 to December 2017. Our search strategy focused on Left Ventricle GLS and adult literature.

Inclusion criteria were human randomized trials, prospective or retrospective observational cohort studies which reported mortality in patients with sepsis, severe sepsis, and/or septic shock utilizing speckle tracking GLS. We have excluded case reports, case series, case-control studies, studies that utilized non-speckle tracking echocardiography methods and studies for which a 2 × 2 table between GLS and mortality could not be constructed by usage of published data. The final results did not include gray or intermediary material. The final study inclusions were based on consensus of 2 reviewers. The third independent reviewer (D.T.) served as the expert referee in case of disagreement. Details of search strategy were included in supplement Tables S1–S3.

The heterogeneity of data in terms of GLS acquisition platforms, proprietary algorithms used for GLS interpretation and patient heterogeneity precludes the combination of data utilizing meta-analysis methods. Hence, we conducted a review of literature.

Out of the initial 191 human studies identified on screening, 8 studies were deemed suitable for analysis and relevant clinical, echocardiographic and outcome, mortality data was tabulated.

## 3. Results

To further assess the role of GLS in sepsis and sepsis-related cardiomyopathy, we tabulated available relevant GLS studies in sepsis cardiomyopathy by performing a literature search for GLS and/or sepsis and/or cardiomyopathy and highlight the following: (Table 1, Table 2 and Table 3).

We tabulated 8 studies including 846 subjects with severe sepsis and/or septic shock. With the exception of 1 study [11] which utilized the Sepsis 3 definition [12]; all others were based on Sepsis 2 criteria [13]. Significant heterogeneity in subjects exists: 5 studies included septic shock patients, 2 studies [14,15] included patients with both severe sepsis and/or septic shock (Table 1).

Of the 846 patients included in these studies, 297 (35.1%) were eliminated from further analysis by various exclusion criteria (Table 2) illustrating the difficulties in quality image acquisition in a timely manner in this set of severely ill patients. With a single exception [14], all studies involved only a single center site.

## 4. Discussion

Several recent studies and a review/meta-analysis [21] shed light on the important question; is GLS is a better predictor of mortality in sepsis cardiomyopathy than the traditional parameter; LV ejection fraction. In their meta-analysis [21], the authors pooled available and eligible observational studies that included 794 patients with severe sepsis and/or septic shock. The pooled data, stratified by survivors/non-survivor, showed that GLS measurements were strongly associated with survival (standard mean difference (SMD) −0.26; 95% confidence interval (CI) −0.47, −0.04; *p* = 0.02) while in contrast, LV ejection fraction was found not to be a predictor of mortality.

Before conclusions can be drawn about GLS’s utility and prognostic value, caution should be applied in interpreting the results of the meta-analysis [21] in view of the heterogeneity, observational nature of the component studies, especially differences in image acquisition platforms and inter-vendor variability in speckle tracking algorithms.

Another recent systematic review [22] which analyzed total of 455 patients [23] did not combine the data by usage of meta-analysis methods citing significant methodological and statistical differences between the studies which concurs with our concerns. The current review included studies published in later half of 2017 and not restricted to studies published in English. We highlight the current inherent limitations of GLS; arising from proprietary differences in image acquisition platforms and inter-vendor variability in speckle tracking algorithms.

At present no accepted GLS thresholds that define sepsis cardiomyopathy exist. The traditionally used abnormal threshold of −20% to define Left ventricular dysfunction may not apply to the setting of sepsis cardio myopathy in the critically ill population [24] and ASE-chamber quantification guideline [5]. The common observation in current literature in terms of predicting outcome is that the lower (less negative) the value for GLS, the worse the outcome, especially among patients “normal” LV ejection fractions.

Practical difficulties in obtaining reliable and timely bedside measurements of GLS exist.

Issues with standardization [24], Inter-Vendor differences [25,26], incorporation/availability of required software in point of care ultrasound machines, training of bedside ICU providers on measurements of GLS, the limited echo windows which may be available in ICU subjects and time constraints to measure GLS (currently off-line for the most part) in the critically ill subset of patients should be recognized and need to be overcome to make this assessment more robust. The current review is not an exhaustive, comprehensive literature search and intends to serve the purpose of outlining the current body of knowledge and limitations of GLS in the context of sepsis cardiomyopathy.

## 5. Conclusions

As the literature on this topic continues to evolve and data accumulates on the value of GLS in sepsis and sepsis cardiomyopathy, the time has arrived to conduct prospective, multi-center investigations to define the role of GLS and potential prognostication thresholds in the management of these critically-ill patients. As such studies are designed, investigators need to take into account the limitations of the prior studies as listed above. Efforts towards future standardization of GLS measurements as being proposed by European Association of Cardiovascular Imaging (EACVI) American Society of Echocardiography (ASE) strain standardization task force will potentially apply to future studies and bring the much needed standardization, facilitation of data pooling and wider applicability of GLS to critical care patients. Future studies should be done utilizing Sepsis 3 definition [16], GLS measurements at pre-defined time points during resuscitation and exploring a combination of patient centric outcome measures (such as duration of mechanical ventilation, duration of pressors, ICU stay, volume status) in addition to mortality outcomes. Until such studies are performed, GLS remains just another tool in our toolbox in the assessment of these complex, critically-ill patients.

In summary, the parameter of GLS heralds an exciting but evolving new era and appears to represent a significant advance in the field of sepsis cardiomyopathy.

## Figures and Tables

**Table 1:** Description of studies including design, inclusion criteria, subjects and imaging platforms/software.

Study	Setting	Geography	Study Design	Study Period	Total Patients	Excluded Pts	No. of Centers	Inclusion Criteria	Primary Outcome	Secondary Outcomes	Cut off Threshold GLS	Echo Machine	Software	Timing	Operator	r^2^ Intra	r^2^ Inter	Ventilator	Shock
											(%)							(%)	(%)
Chang et al., 2015 [16]	University Hospital ICU	Taiwan	Prospective observational	January 2011–June 2013	111	25	1	Septic shock	ICU mortality	Hospital mortality	−13	GE Vivid-I or Q	EchoPAC	<24 h	2 blinded	0.88	0.94		
De Geer et al., 2015 [17]	University Hospital mixed ICU	Sweden	Prospective observational	October 2012–September 2014	44	7	1	Septic shock	ICU mortality	30 days, 90 days mortality	−15	GE Vivid E9	EchoPAC 112	<24 h	1	0.92		84	
Landesberg et al., 2014 [18]	Tertiary academic institute	Israel	Prospective observational	April 2009–March 2011	106	14	1	Severe sepsis and septic shock	hs-cardiac troponin elevation	Hospital mortality		Philips IE33	Philips Qlab 8.1	<24 h	2 blinded			100	
Orde et al., 2014 [19]	Tertiary academic center	USA	Prospective observational	August 2007–January 2009	60	13	1	Severe sepsis and septic shock	30 days mortality	6 months mortality	−17	GE Vivid 7	Syngo Velocity Vector	<24 h	3	0.9 ± 0.9	0.8 ± 0.5	65	
Palmeieri et al., 2015 [20]	ED-HDU academic center	Italy	Prospective observational	October 2012–April 2015	115	34	1	Sepsis and septic shock	28 days mortality	7 days mortality		Philips IE33	Philips Qlab 8.1	<24 h	3 blinded	0.9	0.82	0	39
Zaky et al., 2016 [15]	Tertiary care center any ICU	USA	Retrospective observational	January 2008–December 2011	54	43	1	Sepsis and/or septic shock	In-hospital mortality	Mechanical ventilation, ICU & hospital stay	−15	Philips IE33	Philips Qlab 4.1	<7 days	5	0.83	0.84		
Lanspa et al., 2017 [14]	Tertiary academic centers (2 hospitals, 3 ICUs)	USA	Prospective observational	October 2012–November 2015	298	154	2	Severe sepsis or septic shock	In-hospital mortality, 28 days mortality	Organ failure free days	−17	Philips IE33 or CX50	Image Arena	<24 h				31	39
Yang et al., 2017 [11]	Academic center	China	prospective observational	January 2016–April 2017	58	7	1	Septic shock per sepsis 3	28 days mortality			GE Vivid-Q	EchoPAC	<24 h, day 1,3,7,14	2				100

These published studies utilized different strain analysis software and echo imaging platforms (Table 1): Philips Qlab 8.1^®^ was utilized in 3 studies (*n* = 352), EchoPACS^®^ in 3 studies (*n* = 213), Image Arena^®^ in 1 study (*n* = 298) and Syngo Velocity Vector^®^ (*n* = 60). Philips IE 33^®^ was used for Image acquisition in 4 studies (*n* = 573) and GE Vivid^®^ in 4 studies (*n* = 273). The end points reported were heterogeneous and variable (Table 3).

**Table 2:** Exclusion Criteria.

Study	Exclusion Criteria
Chang et al., 2015 [16]	none
De Geer et al., 2015 [17]	death < 24 h, treatment limitations, no consent, Heart Failure, Ischemic Heart Disease
Landesberg et al., 2014 [18]	Moderate mitral/aortic disease, poor windows, Atrial Fibrillation, arrhythmia, Regional Wall Motion Anamoly
Orde et al., 2014 [19]	pregnancy, congenital Heart Disease, poor image quality, prosthetic valves, cardiomyopathy, moderate or severe valve disease
Palmeieri et al., 2015 [20]	poor windows, greater than moderate aortic or mitral valve disease
Zaky et al., 2016 [15]	Age < 18 years, Atrial Fibrillation, LVEF < 40%, valve disease, valve replacement, ICDs, poor Echo views
Lanspa et al., 2017 [14]	echo > 24 h, poor image quality
Yang et al., 2017 [11]	Myocardial Infraction, congenital, valvular heart disease, hospitalization < 24 h, malignancy, liver, kidney failure, pericardial effusion, advanced malignancy, poor image quality

These published studies utilized different strain analysis software and echo imaging platforms (Table 1): Philips Qlab 8.1^®^ was utilized in 3 studies (*n* = 352), EchoPACS^®^ in 3 studies (*n* = 213), Image Arena^®^ in 1 study (*n* = 298) and Syngo Velocity Vector^®^ (*n* = 60). Philips IE 33^®^ was used for Image acquisition in 4 studies (*n* = 573) and GE Vivid^®^ in 4 studies (*n* = 273). The end points reported were heterogeneous and variable (Table 3).

**Table 3:** Outcomes.

Study	ICU Non Survivor GLS	ICU Survivor GLS	Hospital Non Survivor GLS	Hospital Survivor GLS	28 Days Non Survivor GLS	28 Days Survivor GLS	30 Days Non Survivor GLS	30 Days Survivor GLS	90 Days Non Survivor GLS	90 Days Survivor GLS	6 Months Non Survivor GLS	6 Months Survivor GLS	Abnormal GLS Hospital Mortality	Abnormal GLS Hospital Mortality	Normal GLS Hospital Mortality	Abnormal GLS 28 Days Mortality
	Mean ± SD in %												Alive n (%)	Dead n (%)	Dead n (%)	Dead n (%)
Chang et al., 2015 [16]	−11.8 ± 4.5	−15 ± 3.6	−12.4 ± 4.9	−14.9 ± 3.4												
De Geer et al., 2015 [17]							−15 (−19.7 to −11)	−17.2 (−20 to −13)	−14.7 (−19 to −10.6)	−17.4 (−20.5 to −13.6)						
Landesberg et al., 2014 [18]			−12.3 ± 3.6	−13.7 ± 2.7												
Orde et al., 2014 [19]							−14.6 ± 4.3	−13.92 ± 4.2			−14.28 ± 4.6	−14 ± 4				
Palmeieri et al., 2015 [20]					−9.1 ± 3.6	−10.8 ± 3.2										
Zaky et al., 2016 [15]													24 (80)	12 (66.7)		
Lanspa et al., 2017 [14]														47 (22)	31 (17)	54 (25)
Yang et al., 2017 [11]					−15.98 ± 1.41	−17.66 ± 1.22										

These published studies utilized different strain analysis software and echo imaging platforms (Table 1): Philips Qlab 8.1^®^ was utilized in 3 studies (*n* = 352), EchoPACS^®^ in 3 studies (*n* = 213), Image Arena^®^ in 1 study (*n* = 298) and Syngo Velocity Vector^®^ (*n* = 60). Philips IE 33^®^ was used for Image acquisition in 4 studies (*n* = 573) and GE Vivid^®^ in 4 studies (*n* = 273). The end points reported were heterogeneous and variable (Table 3).

## Supplementary Materials

The following are available online at http://www.mdpi.com/2227-9032/7/1/5/s1, Table S1: Description of studies including design, inclusion criteria, subjects and imaging platforms/software, Table S2: Exclusion Criteria, Table S3: Outcomes.
